# Green synthesis of silver and iron oxide nanoparticles mediated photothermal effects on *Blastocystis hominis*

**DOI:** 10.1007/s10103-024-03984-6

**Published:** 2024-01-22

**Authors:** Shaimaa M. I. Alexeree, Hanan M. Abou-Seri, Hala E. Shams EL-Din, Doaa Youssef, Marwa A. Ramadan

**Affiliations:** 1 Department of Laser Application in Metrology, Photochemistry, and Agricultural, National Institute of Laser Enhanced Science, Cairo University, Giza, Egypt; 2 Department of Parasitology, Faculty of Medicine, Ain Shams University, Cairo, Egypt; 3 Department of Engineering Applications of Lasers, National Institute of Laser Enhanced Science, Cairo University, Giza, Egypt

**Keywords:** Metal nanoparticles, Photothermal therapy, Ag NPs, Fe_3_O_4_ NPs, Parasite, Superpixel

## Abstract

The evolution of parasite resistance to antiparasitic agents has become a serious health issue indicating a critical and pressing need to develop new therapeutics that can conquer drug resistance. Nanoparticles are novel, promising emerging drug carriers that have demonstrated efficiency in treating many parasitic diseases. Lately, attention has been drawn to a broad-spectrum nanoparticle capable of converting absorbed light into heat via the photothermal effect phenomenon. The present study is the first to assess the effect of silver nanoparticles (Ag NPs) and iron oxide nanoparticles (Fe_3_O_4_ NPs) as sole agents and with the combined action of the light-emitting diode (LED) on *Blastocystis hominins* (*B. hominis*) in vitro. Initially, the aqueous synthesized nanoparticles were characterized by UV-Vis spectroscopy, zeta potential, and transmission electron microscopy (TEM). The anti-blastocyst efficiency of these NPs was tested separately in dark conditions. As these NPs have a wide absorption spectrum in the visible regions, they were also excited by a continuous wave LED of wavelength band (400–700 nm) to test the photothermal effect. The sensitivity of *B. hominis* cysts was evaluated using scanning laser confocal microscopy whereas the live and dead cells were accurately segmented based on superpixels and the *k*-mean clustering algorithm. Our findings showed that this excitation led to hyperthermia that induced a significant reduction in the number of cysts treated with photothermally active NPs. The results of this study elucidate the potential role of photothermally active NPs as an effective anti-blastocystis agent. By using this approach, new therapeutic antiparasitic agents can be developed.

## Introduction

*Blastocystis hominis* (*B. hominis*) is the most prevalent intestinal colonizing parasite that infects at least one billion people worldwide. Its prevalence rates vary between countries as well as between regions in the same country [1]. It may vary between 30 and 76% in developing nations and reach up to 30% in industrialized countries. Recently, several reviews have indicated that higher prevalence in developing countries was due to environmental contamination, poor sanitation, and bad personal hygiene practices [2, 3]. In addition, exposure to domestic animals and fecally contaminated food and water is common [4]. *B. hominis* is an obligatory, unicellular, eukaryotic, anaerobic protozoan. It is the most common gastrointestinal parasite present in the stool samples of humans and animals [5, 6]. Such a polymorphic parasite has four major forms found in the stools and in vitro cultures: vacuolar, granular, amoeboid, and cyst [7]. The life cycle of *B. hominis* is initiated by the cyst as an infective stage. After ingestion, it develops into a vacuolar form in the large intestine. These vacuolar forms are divided into amoeboids or granular by binary fission [8].

The clinical manifestations of *Blastocystis* infection are non-specific and consist of abdominal pain, acute/chronic diarrhea, bloating, nausea, and anorexia. Symptoms might range from mild to moderate to severe acute and chronic events, especially in children and immunocompromised patients [9]. On the other hand, it has been considered as a commensal parasite in humans that can persist with being unable to trigger any diseases [10]. *B. hominis* was assumed to have a vital effect on the intestinal microbiome and has been associated with several diseases such as functional gastrointestinal disorders known as irritable bowel syndrome (IBS), hemorrhagic proctosigmoiditis, inflammatory bowel disease (IBD), and chronic spontaneous urticaria (CSU). The exact pathophysiological mechanism is not yet settled but may be due to an alteration of the intestinal permeability by pro-inflammatory cytokines resulting in visceral inflammation and hypersensitivity [11, 12]. Several methods have been utilized for the diagnosis of *B. hominis*, such as direct microscopic examination, phase contrast microscopy, immunodiagnostics, in vitro cultivation, and molecular analysis [11, 13]. Regarding the treatment of *B. hominis*, which is questioned as all gastrointestinal symptoms are self-limited without complications in many patients, metronidazole is considered the main treatment [13–15]. Since 1976, several in vitro studies have documented the failure of metronidazole to cause complete clearance of *B. hominis*. Consequently, the evolution of parasite resistance to antiparasitic agents has become a serious health issue that necessitates the development of new and efficient antiparasitic agents [16, 17].

Nanoparticles (NPs) are new promising drug carriers, demonstrated to be efficient in treating many parasitic diseases. This potency is related to the ability to overcome constraints such as poor cellular permeability, low bioavailability, nonspecific distribution, and quick drug elimination from the body [18]. Nanomaterials are more important for the emerging fields of nanomedicine, nanobiotechnology, and nanotoxicology [19–22]. Whereas, in the toxicity field, NPs are being utilized as therapeutic tools against pathogenic microorganisms. Therefore, it is essential to study the nanoparticles’ properties and their usage in different biological and medical applications to understand their effect on parasites, bacteria, fungi, etc. [23, 24]. Moreover, the type of stabilizing and capping materials used for preparing NPs is important as it affects their antimicrobial effectiveness [25]. In general, NPs have different features compared to the same bulk materials [26]. Such that the surface-to-volume ratio of NPs increases by decreasing the particle size. The unique size-dependent properties of inorganic nanomaterials are essential in many areas of human activity. They are widely considered a platform for targeted drug delivery, clinical diagnostics, and medical imaging. The most common inorganic nanoparticles used for these purposes are silver, iron oxide, gold, zinc oxide, and titanium.

Metallic nanoparticles show new optical properties, which are not observed in bulk metals [27, 28]. One of these properties is the presence of an absorption band. This band is due to the surface plasmon-oscillation modes of conduction electrons that are coupled through the surface to external electromagnetic fields. Among noble metal nanomaterials, silver nanoparticles (Ag NPs) have received considerable attention owing to their attractive physicochemical properties. In addition, the strong toxicity that silver exhibits in various chemical forms to a wide range of microorganisms is very well known. Ag NPs have recently been shown to be a promising antimicrobial as well as antiparasitic material. A few investigations have shown that different surface stabilizers have important effects on Ag NP cytotoxicity. Due to its great biocompatibility and antipathogenic properties, chitosan (Cs) is utilized as an active ingredient in topical wound materials [29]. Furthermore, several studies have reported that chitosan is considered a good stabilizer for Ag NPs [30, 31]. Moreover, chitosan-coated silver nanoparticles show high effectiveness in killing common gram-positive and gram-negative bacteria, and fungi [32].

Magnetic nanoparticles are the first generation of nanomaterials approved for clinical use. The superparamagnetic properties increased the possibility of developing novel and efficient biomedical applications [33]. These applications targeted drug and gene delivery, magnetic resonance imaging, biosensors, cancer detection and treatment, diagnosis and magnetic field-assisted radiotherapy, and tissue engineering [34]. A common type of iron oxide nanoparticles is magnetite (Fe_3_O_4_), which belongs to the ferrimagnetic class of magnetic nanomaterials. Fe_3_O_4_ NPs are highly advantageous due to their biocompatibility, biodegradability, non-toxicity, and ability to specifically target tissue. Thus, magnetite nanoparticles could represent a novel and efficient direction in the management of infectious diseases [35].

Generally, Ag NPs and Fe_3_O_4_ NPs are easy to synthesize, safe for biomedical applications, and display attractive optical absorption covering the visible and near-infrared (NIR) region. Moreover, they can stand out as functional nanomaterials for photothermal therapy (PTT) [36, 37]. PTT is the most efficient method that has been applied for the treatment of various medical situations including cancer, inflammatory, or microbial diseases. PTT is highly dependent on the excitation of a photosensitizer with a specific band of light that generates vibrational energy. The effect of such a local hyperthermal mechanism induced irreversible damage to the targeted cells by causing protein denaturation, coagulation, cell membrane destruction, and/or bubble formation around NPs upon irradiation [38, 39].

To sum up, silver and iron oxide nanoparticles are biocompatible and non-toxic materials with attractive properties that can manage microbial and parasitic diseases. This indicates that such NPs, as photothermal absorbers, can enhance photothermal therapy by providing a promising treatment method. To the best of our knowledge, no single study exists to explore the effect of photothermally active metal NPs on *B. hominis* in vitro. Therefore, the present work aims to assess the effect of Ag NPs stabilized with chitosan and Fe_3_O_4_ NPs coated with polyethylene glycol (PEG) as sole agents and in combined action with LED at the wavelength band 400–700 nm to evaluate their potential anti-blastocystis activity.

## Materials and methods

### Preparation of samples

#### Stool sample collection

Fresh stool samples were collected from patients attending the Clinical Pathology Laboratory of Ain Shams University Hospitals. Each patient had a history of gastrointestinal symptoms, including diarrhea and abdominal pain, but had no previous prescribed treatment. The stool samples were transferred on the same day of collection to the Parasitology Department, Faculty of Medicine, Ain Shams University, and stored at 4 °C before further analysis. Samples were examined microscopically by wet smear preparation in saline and staining with Lugol iodine to confirm the presence of *B. hominis* infection and exclude any other parasitic infections. Only positive stool samples for *Blastocystis* without co-infection with other parasites were included in the study.

#### Cultivation and subculture

Positive stool samples were cultivated on the same day of collecting samples in 10-mL screw caped culture tubes containing a previously prepared LE medium (National Institute of Health (NIH) modification of Boeck and Drbohlav’s medium), supplemented with 10% bovine serum (Biowest) and antibiotic mixture ready-made of penicillin (10000 U/mL) and streptomycin (10000 μg/mL) (Biowest). The culture tubes were incubated at 37 °C and examined for growth after 24, 48, and 72 h using an inverted microscope ×10 and ×40 magnification lens. They were considered negative for *B. hominis* when no parasite growth was detected after 72 h of culture and incubation. For positive tubes, subculture was done on new culture tubes to maintain the growth every 48 h.

### Synthesis of metal nanoparticles

#### Green synthesis of silver nanoparticles coated with chitosan

Chitosan in flake forms prepared from crab shells (practical grade >85% deacetylated; Brookfield viscosity >200,000 cps), silver nitrate, and acetic acid were purchased from Sigma-Aldrich Chemical Co. Cs was dissolved in a 1% acetic acid solution. Briefly, to prepare silver nanospheres coated with Cs, grade purity was used as the starting material without further purification, and 4 mL of 52 mM silver nitrate Ag NO_3_ was added to 10 mL (6.92 mg/mL) of the chitosan solution, mixed, and stirred until complete homogeneity. Then, the mixture was transferred to a 12.5-mL bottle and allowed to stand for 12 h at 95 ℃. The color of the solution progressed from colorless to light yellow. Finally, the yellowish-brown was obtained within hours after the initiation of the reaction. The silver samples were used directly for the antibacterial activity determination. For measuring the activity of the silver itself, a sample was adjusted at pH = 5 with 1 M NaOH and subjected to the antibacterial activity assays [31].

#### Synthesis of iron oxide nanoparticles coated with polyethylene glycol

The preparation process of Fe_3_O_4_ NPs may lead to considerable changes in the final product’s co-precipitation of Fe salts. Therefore, there are several magnetite synthesis methods to generate controlled sizes and morphologies. The addition of a hydroxide base allows the preparation of Fe_3_O_4_ nanoparticles in a simple way with precise control of size and shape for biomedical applications. The process of co-precipitation was carried out at pH = 10 and can be represented by the following chemical equation: Fe[H_2_O]_6_^2+^ + Fe[H_2_O]_6_^3+^ + NaOH = Fe(OH)_2_ + Fe(OH)_3_ **→** Fe_3_O_4_**↓.** Magnetite nanoparticles were prepared using 100 mL of a 3% PEG (Sigma-Aldrich, MW=8000) solution in deionized water. The PEG solution was bubbled with nitrogen gas for 30 min. Then, 1.7 g of iron chloride anhydrous (Sigma-Aldrich, MW =162.21) and 2 g of ammonium iron sulfate hexahydrate (Sigma-Aldrich) were dissolved in the PEG solution with stirring. Three M NaOH (Alfa Aesar) was added dropwise into the mixture under nitrogen gas with vigorous stirring. The brown precipitate formed and turned black. The magnetite nanoparticles were collected with a magnet, washed several times with deionized water, and dried in a vacuum oven [40].

### Characterization of Ag and Fe_3_O_4_ nanoparticles

The spectral absorption of the explored metal nanoparticles was measured using a double-beam UV-Vis spectrophotometer (Cary 5000, Agilent Technologies, Santa Clara, CA, USA). Their morphology was imaged using a high-resolution transmission electron microscope (HRTEM, Tecnai, G20, FEI, Almelo, Netherlands) operating at an accelerating voltage of 200 kV. The drops of dilute prepared nanomaterial solutions were deposited on a carbon-coated copper grid and left to dry at room temperature before the examination by TEM. In addition, the electrokinetic potential (zeta potential) was measured with a zeta sizer analyzer (Nano ZS, Malvern Instruments, Malvern, UK) based on an electrophoretic light scattering technique.

### Photothermal activation of Ag and Fe_3_O_4_ nanoparticles

Inoculum size was initially assessed by counting the number of live parasites using a Neubauer cell counting chamber after staining with a 0.4% Trypan blue solution (a viability indicator). A parasite inoculum of size 40×10^4^ parasites/mL from culture during the logarithm phase was considered suitable for assessment. After calculation of the inoculum volume required, the parasites were introduced into a set of culture tubes containing LE medium and three different concentrations of either Ag NPs or Fe_3_O_4_ NPs to evaluate the parasite sensitivity in the dark condition. Similarly, 2 mL of each parasitic suspension was seeded in each well of a 6-well plate with a flat bottom. The cells were photo-irradiated with broad-band visible light (400–700 nm) using an LED lamp as a light source (10 W, Wellmax, China) [41]. Culture tubes were used in triplicate for every concentration of each nanoparticle and the non-treated control. The summary of the groups used in the present work is displayed in Table 1.

**Table 1:** Analysis of the studied groups within the present work

Groups	Description
Group 1 (L− Ag− IO−)	Cells were not exposed either to Ag NPs, Fe_3_O_4_ NPs, or light
Group 2 (L+ Ag− IO−)	Cells were exposed to four different doses of visible light 1.8, 3.5, 5.3, and 10.6 J/cm^2^ in tubes without Ag NPs or Fe_3_O_4_ NPs
Group 3 (L− Ag+)	Cells were incubated with different concentrations of Ag NPs (10, 20, and 30 μM) in dark conditions
Group 4 (L+ Ag+)	Cells were pre-incubated with Ag NPs for 20 min followed by irradiation with visible light for 5 min
Group 5 (L− IO+)	Cells were incubated with different concentrations of Fe_3_O_4_ NPs (10, 20, and 30 μM) in dark conditions
Group 6 (L+ IO+)	Cells were pre-incubated with Fe_3_O_4_ NPs for 20 min followed by irradiation with visible light for 5 min

*L* light irradiation, *Ag* silver nanoparticles (Ag NPs), *IO* magnetite nanoparticles (Fe_3_O_4_ NPs)

### Blastocystis viability via confocal laser scanning microscopy

The cell concentration and viability of *Blastocystis* cysts within each analyzed group were assessed by an acridine orange/propidium iodide (AO/PI) double staining mixture. This staining dye can quantify the morphological apoptotic changes in the treated cells and, hence, differentiate live cells from dead ones by utilizing a fluorescence microscope [42]. The AO dye can penetrate the cell membranes of viable and dead cells, staining their nuclear DNA and exhibiting green fluorescence under blue excitation [43], while the PI dye penetrates only decomposed cell membranes and stains the denatured DNA emitting red fluorescence under green excitation [43, 44]. After 24 h of incubation at 37 °C, counting the number of viable cells was done the same way as before. Subsequently, each group was washed three times with phosphate buffer (PBS), PH = 8, and then centrifuged. The supernatant was discarded, and the pellet was redispersed in 100 µL of PBS containing 10 µL of an equal volume AO/PI mixture. Then, the *B. hominis* cells were incubated with the mixture for 30 min in the dark. About 10 µL of each sample was examined by a ×63 confocal laser scanning microscope (CLSM, LSM 710, Carl Zeiss, Germany). The images were collected at excitation wavelengths of 458 nm for AO and 514 nm for PI. Under the excitation of 458 nm, the green fluorescence of AO emitted by dead cells was absorbed by PI generating weak green emission.

### Image processing for cell extraction

Automatic cell segmentation and clustering of the fluorescence images is a challenge. For example, morphological watershed-based algorithms were employed to separate different clustered nuclei [45, 46]. After weighting each pixel concerning its near boundaries’ curvature information, a distance transform and watershed method was used for the extraction of nuclei [47]. Nuclei segmentation from confocal images was performed employing gradient flow tracking and locally adaptive thresholding [48]. Besides, automatic threshold selection methods for the CLSM images of biofilms were established [49, 50].

In this study, the color features of the live and dead cells based on superpixels and *k*-means clustering were utilized to segment and classify the live and dead cells. Different superpixel approaches have been suggested and applied in computer vision algorithms as a key intermediate representation of an image to be broken into perceptually meaningful superpixel regions. As a result, segmentation and classification can be done over regions instead of the full image, providing high-quality results. An efficient superpixel approach should be fast and simple, maintain memory size and computation accuracy, and adapt well to image boundaries [51–54].

Since the proposed method for the extraction of live and dead cells was based on their color features, the collected CLSM images were converted into the CIELAB colorspace. The presumed reason is that the CIELAB is a perceptually uniform colorspace that depicts all visible colors to the human eye [50, 51]. It comprises three components, ${l}^{*}$, ${a}^{*}$, and ${b}^{*}$. Whereas ${l}^{*}$ represents the luminance, while ${a}^{*}$ and ${b}^{*}$, respectively, define the amounts of red-green and yellow-blue tones. After such a preprocessing step, the fast superpixel approach called *adaptive simple linear iterative clustering* (ASLIC) [51] was applied to partition the image into superpixel regions employing the *k*-means clustering method for a fixed number of iterations. Then, we started with an initial estimate of the number of superpixels, *k*. Since the *k*-means method tries to minimize the average square distance between pixels in the same cluster [55], the distance measure, *D*, was defined as follows [51]:

1 $$D=\sqrt{{{d}_{c}}^{2}+{({~}^{C}\!\left/ \!{~}_{S}\right.)}^{2} {{d}_{s}}^{2}}$$

2 $${d}_{c}=\sqrt{{({{l}^{*}}_{j}-{{l}^{*}}_{i})}^{2}+{({{a}^{*}}_{j}-{{a}^{*}}_{i})}^{2}+{({{b}^{*}}_{j}-{{b}^{*}}_{i})}^{2}}$$

3 $${d}_{s}=\sqrt{{({x}_{j}-{x}_{i})}^{2}+{({y}_{j}-{y}_{i})}^{2}}$$

4 $$S=\sqrt{N/k}$$

where ${(x}_{i},{y}_{i})$ and $({x}_{j},{y}_{j})$ are the coordinate values of pixels $i$ and $j$, respectively. ${d}_{c}$ and ${d}_{s}$ are the CIELAB space and Euclidean distances, respectively. *S* is the average distance between two cluster centers, $N$ is the total number of image pixels, and *C* is the compactness that weighs the relationship between the color and spatial distances. So by iteration, the ASLIC algorithm updates the values of *S* and *C* for each cluster, utilizing the maximum color and spatial distances from the preceding iteration [51]. Then, the mean color of the intensity distribution within each superpixel region was computed to quantify the superpixel regions and generate a label image. Further analysis by applying another *k*-means clustering to the label image was performed, by which the CLSM images were accurately segmented into three clusters, live cells, dead cells, and background.

### Statistical analysis

Data were reported as mean ± standard deviation (SD) for quantitative variables of triplicate determinations, and comparisons between the different means of the studied groups were done using the Student *t*-test to assess the statistical significance. The values of *p* < 0.05 were considered statistically significant.

## Results and discussion

### Synthesis and characterization of Ag and Fe_3_O_4_ nanoparticles

**Fig. 1:**
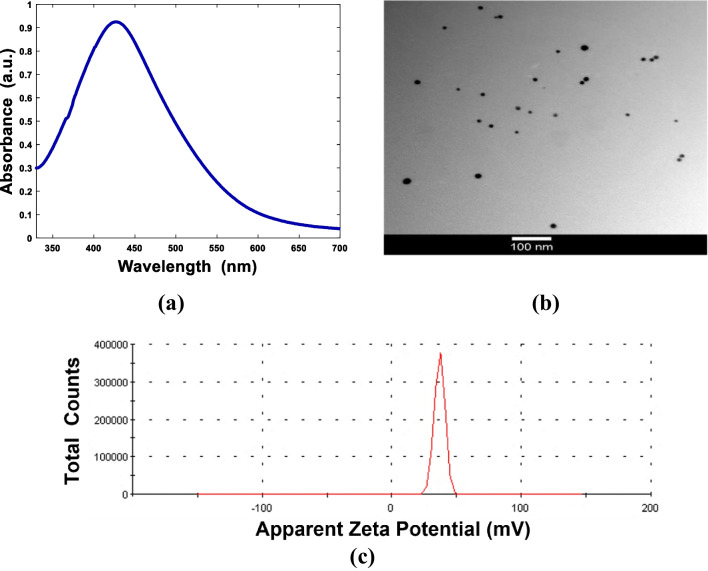
Characterization of Ag NPs coated with Cs. **a** UV-Vis absorption curve, **b** TEM image (magnification 100 nm), and **c** zeta potential

Silver nanoparticles were synthesized by reducing silver nitrate salts with non-toxic and biodegradable chitosan at given temperatures. The typical UV-Vis absorption spectrum of the resulting solutions is shown in Fig. 1a. The plot displays that the characteristic surface plasmon resonance (SPR) band is centered at about 425 nm. This band represents the formation of silver nanoparticles. The appearance of a yellowish-brown color also supported the formation of Ag nanoparticles. Such results are consistent with the previous reports on the fabrication of gold nanoparticles with chitosan as both a stabilizing and reducing agent [56]. The TEM result indicates the spherical shape of Ag NPs with a particle size in the range of 10 nm, distributed homogeneously in the Cs matrix (Fig. 1b). The average surface zeta potential of Ag coated with Cs was approximately 36.7 mV, as shown in Fig. 1c. This value can greatly influence their stability to form a stable solution in water using electrostatic repulsion between the particles. In addition, it facilitated its absorption by a cellular membrane and made them good candidates to be used for therapy and imaging while coated with Cs.

**Fig. 2:**
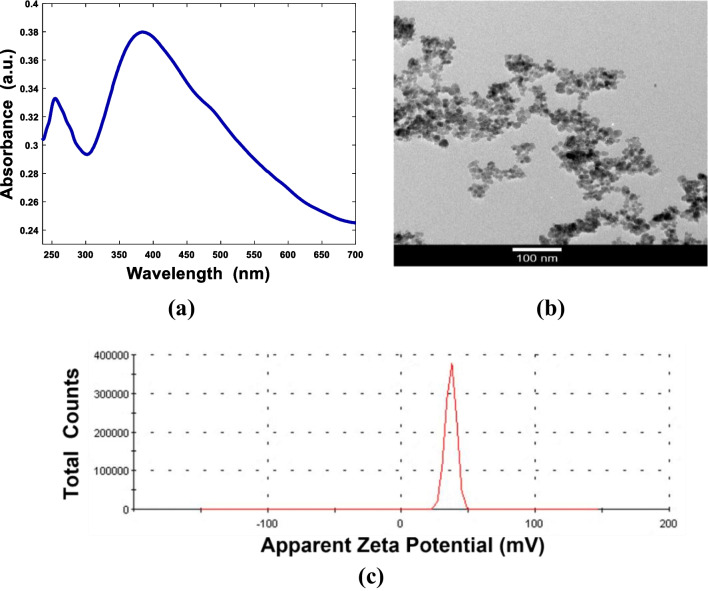
Characterization of Fe_3_O_4_ NPs coated with PEG. **a** UV-Vis absorption curve, **b** TEM image (magnification 100 nm), and **c** zeta potential

The successful synthesis of the magnetite nanoparticles was confirmed by the UV-Vis spectrum (Fig. 2a). The solution had a dark background color with a broad absorption band from UV to NIR. A representative TEM image of polymer-coated magnetite nanoparticles is shown in Fig. 2b. It displays the production of a large quantity of nearly uniform monodispersed nanospheres of iron oxide. The size of the magnetite was about 10 nm. The surface of magnetite nanoparticles was mostly negatively charged due to coating with PEG, as observed from zeta potential measurements (Fig. 2c). The average surface potential was −6 mV. This charge value enabled it to form a stable solution in the water. Such a finding was related to the small crystallite sizes of the magnetite particles. The superparamagnetic properties of iron oxide nanoparticles make them good candidates to be used in many biomedical applications [18, 40].

### Design the enhanced therapy of the antiparasitic pathway

Although some studies reported the efficiency of PTT on pathogenic organisms, no previous studies represented the photothermal effect of LED combined with metal nanoparticles on *B. hominis*. Our design was prepared to obtain a synergetic, enhanced antiparasitic effect. This occurred by combining the intrinsic antiparasitic activity of metallic nanoparticles with their photothermal effect after irradiation with the LED (400–700 nm) to induce local hyperthermia that caused cell death. Consequently, the synergistic effect of metal nanoparticles (Ag NPs or Fe_3_O_4_ NPs) with the LED (400–700 nm) irradiation was able to induce an anti-blastocystis effect.

#### The effect of the light emitted diode on B. hominis

**Fig. 3:**
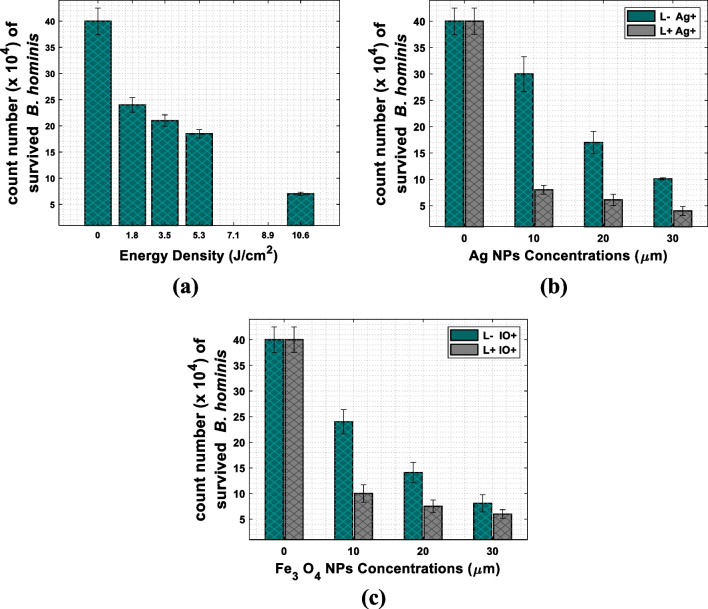
Histogram plots of the count number of the survived *B. hominis* relative to the control group (L− Ag− IO−). **a** After irradiation (L+ Ag− IO−) with four different irradiation doses (1.8, 3.5, 5.3, and 10.6 J/cm^2^), **b** in the presence of three different concentration of Ag NPs at dark (L− Ag+) as well as in the presence of light (L+ Ag+), and **c** in the presence of three different concentrations of Fe_3_O_4_ NPs at dark (L− IO+) as well as in the presence of light (L+ IO+). Error bars represent the standard deviation from three different experiments

Four different irradiation doses (1.8, 3.5, 5.3, and 10.6 J/cm^2^) were applied to *B. hominis* cells in the absence of nanoparticles (L+ Ag− IO−). These doses were used to study the phototoxicity of the LED on *B. hominis* (Fig. 3a). Initially, there was no significant reduction in the *B. hominis* cell count in comparison to the control. However, the decline was observed to be statistically significant starting from the irradiation dose of 3.5 J/cm^2^. The irradiation dose of 1.8 J/cm^2^ was selected as a candidate for the photothermal activation of Ag NPs and Fe_3_O_4_ NPs for further analysis. Whereas, five groups were analyzed: control (untreated), Ag NPs, photothermally active Ag NPs, Fe_3_O_4_ NPs, and photothermally active Fe_3_O_4_ NPs. Three concentrations of Ag NPs or Fe_3_O_4_ NPs (10, 20, and 30 µM) were applied alone and in combination with 1.8 J/cm^2^ doses of the LED (400–700 nm).

#### The photothermal effect of Ag NPs on B. hominis

The responses of *B. hominis* incubated with Ag NPs in dark conditions (L− Ag+) using different concentrations were investigated. The results indicated that the incubation of *B. hominis* with Ag NPs at 10 µM posed a non-statistically significant reduction concerning the untreated group (L− Ag− IO−) (25%). Furthermore, by increasing Ag NP concentration to 30 µM, the cell count was reduced by 75% in comparison to the control (Table 2 and Fig. 3b). This result demonstrated the evident effect of the Ag NPs on the parasitic cell count in the dark [44].

**Table 2:** Effect of Ag NPs or Fe_3_O_4_ NPs with and without photothermal effect on the in vitro count of *B. hominis* (cells ×${10}^{4}$) with different concentrations after 24 h of incubation

Group (dosage of treatment)	Ag NPs		Fe_3_O_4_ NPs	
	Dark	Light	Dark	Light
	Mean ± SD (growth inhibition $\text{\%}$)			
Untreated	40±2.5	40±2.5	40±2.5	40±2.5
10 μM	30±3.3 (40%)	8±0.85* (80%)	24±2.35 (40%)	10±1.7* (75%)
20 μM	17±2.1* (57%)	6.1±1.05* (85%)	14.1±2* (65%)	7.5±1.25* (81%)
30 μM	10.1±0.2* (75%)	4±0.85* (90%)	8.1±1.65* (80%)	6±0.85* (85%)

Values are expressed as mean ± SD

*Statistically significant difference compared to untreated ($p<0.05$)

Percent of growth inhibition = ($a-b/a$), where “$a$” is the mean number of parasites in NTC and “$b$” is the mean number of parasites in treated cultures

This inhibitory effect of Ag NPs is in line with a few other studies. These studies used different concentrations of Ag NPs on *B. hominis* in vitro. The result reported a higher significant reduction in *B. hominis* cell count than that detected for Metronidazole [24]. Besides, Ag NPs showed cytotoxic effects on many other parasites, such as *Giardia*, *Toxoplasma*, *Plasmodium*, and even insect larvae [39]. In the same context, Allahverdiyev et al. [57] reported the anti-Leishmanial effects of Ag NPs on *Leishmania tropical* parasites and its enhanced inhibitory effect by exposure to ultraviolet rays. The authors owed these anti-Leishmanial effects to the wide surface area, the small size of Ag NPs, and their ability to produce reactive oxygen species. Moreover, the antibacterial activity of Ag NPs has been documented against *Escherichia coli*, *Staphylococcus aureus*, and *Pseudomonas aeruginosa* [58].

A further statistically significant drop in *B. hominis* cell count was noted upon utilizing the photothermally active Ag NPs (L+ Ag+). This effect was enhanced by a further increase in the concentrations of Ag NPs (Table 2 and Fig. 3b). In addition, the small size of Ag NPs (10 nm) had a stronger effect on the *B. hominis* cells [25]. These results agree with the invention of [34].

#### The photothermal effect of Fe_3_O_4_ NPs on B. hominis

Iron oxide nanoparticles are widely used in biomedical applications. Yet, no previous studies represented the dark toxicity as well as the photothermal effect of magnetite Fe_3_O_4_ NPs on *B. hominis* cells in vitro. Initially, the experiment of the dark cytotoxicity indicated a statistically significant reduction of the parasitic count at concentrations of 20 and 30 µM for Fe_3_O_4_ NPs (L− IO+) in comparison to the untreated group (L− Ag− IO−). Drawing on these conclusions, the photothermal effect experiments were applied for the three concentrations of 10, 20, and 30 µM of Fe_3_O_4_ NPs, as they had a satisfactory effect on the parasitic count in the dark. At the combination of Fe_3_O_4_ NPs with LED 1.8 J/cm^2^ dose at the wavelength band (400–700 nm) (L+ IO+), *B. hominis* showed a statistically significant reduction in cell count relative to the untreated group (Table 2 and Fig. 3c). These results demonstrated the capability of Fe_3_O_4_ NPs as an efficient photothermal agent for antiparasitic purposes.

In agreement with an earlier study [59], Fe_3_O_4_ NPs were utilized in thermotherapy for leishmaniasis in vitro, known as magnetic hyperthermia, to target amastigotes in their intracellular niche. Besides, surface-coated iron oxide nanoparticles were used to enhance the efficacy of Artesunate, the anti-malarial drug, by killing late ring stages of *Plasmodium falciparum*, 8- to10-fold and 5-fold in vivo and in vitro, respectively [60].

### The suggested mechanism of action

The accurate mechanisms postulated for the antipathogenic effects of metal NPs are still being studied. The mechanism suggested for the metal nanoparticles combined with photothermal activation might be related to the enhanced release of ions (Ag^+^ or Fe_3_O_4_^+^) from Ag NPs or Fe_3_O_4_ NPs, respectively, by rising temperature. Moreover, the permeability of the parasitic cell membrane might be disrupted by these ions. Furthermore, many studies have detected that NP force induces holes and gaps in the pathogenic organism’s cell membrane, which leads to cell fragmentation as well as cell death [58]. Otherwise, there was a possibility of oxidative stress occurring through the generation of reactive oxygen species (ROS) on the surface of NPs. Eventually, more damage occurs in the parasitic cell, leading to cell death [61].

**Fig. 4:**
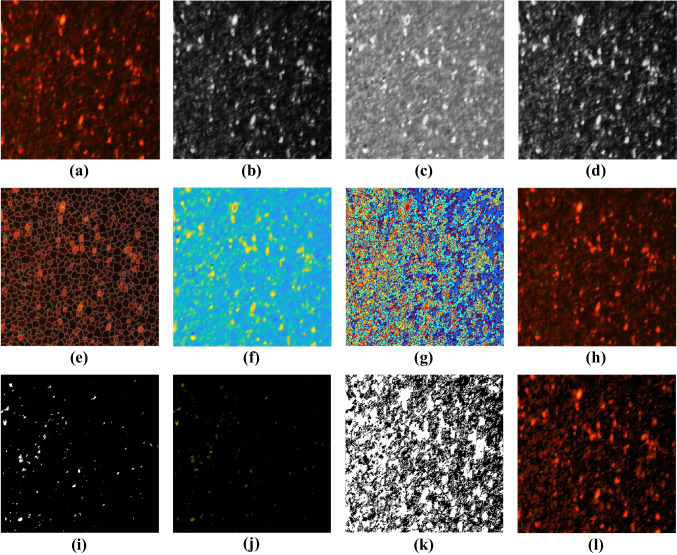
Extraction of live and dead cells from CLSM images. **a** CLSM image collected from Fe3O4 NPs + light group, **b**–**d** ${l}^{*}$, ${a}^{*}$, and ${b}^{*}$ components of the CIELAB colorspace, respectively, **e** CLSM image with superimposed superpixel regions’ boundaries, **f** mean color of the intensity distribution within each superpixel region, **g** label image, **h** result of applying *k*-means clustering on the label image, **i**, **j** extracted binary and color live cells, respectively, and **k**, **l** extracted binary and color dead cells, respectively

**Fig. 5:**
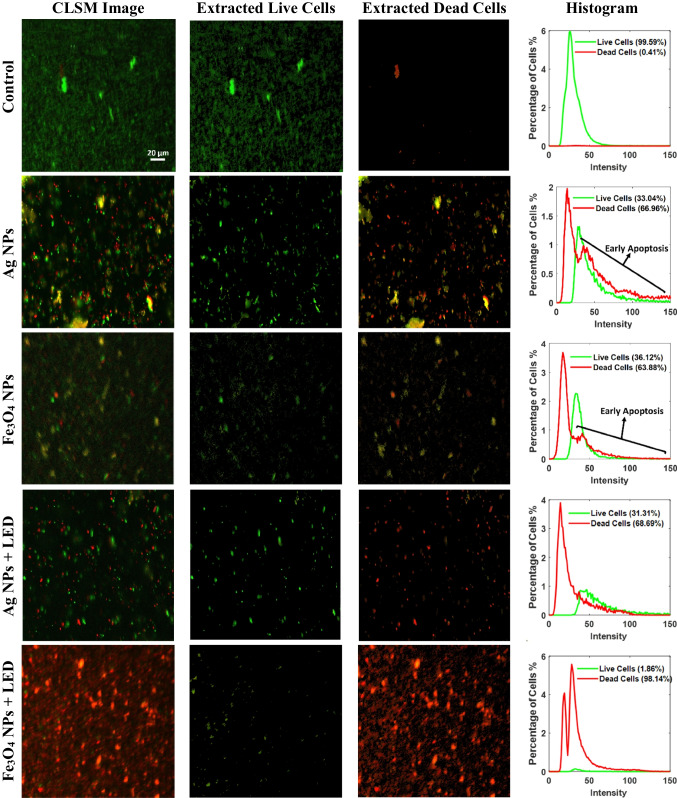
Confocal imaging of live and dead *B. hominis* cells shows the control, Ag NPs (L− Ag+), Fe_3_O_4_ NPs (L− IO+), Ag NPs + light (L+ Ag+), and Fe_3_O_4_ NPs + light (L+ IO+) groups. Acridine orange (AC)-stained cells represent the live cells (green), while the propidium iodide (PI) stained the dead cells (red). Scale bars equal to 20 µm

To investigate the antiparasitic mechanism of the Ag NPs, Ag NPs, Fe_3_O_4_ NPs, and Fe_3_O_4_ NPs combined with LED, the *B. hominis* cyst viability was assessed using CLSM. The two fluorescent nucleic acid dyes, acridine orange and propidium iodide, were used to stain the *B. hominis* cells. The stained *B. hominis* cells were visualized under the CLSM to assess the viability and membrane integrity of the parasite both in the dark and after photothermal activation. Then, a method based on the color features of the live and dead cells employing superpixels and *k*-means clustering was proposed to segment and classify the live and dead cells (see the “Image processing for cell extraction” section). A stepwise example showing the segmentation process is presented in Fig. 4. The results of the extracted live cells and dead cells, as well as histograms stating the percentage of cells showing the proportion of apoptosis, are presented in Fig. 5.

It can be observed that the control group exhibited almost entirely green-colored cells stained with AC, which could be internalized in the live cells. On the other side, the cells treated in the dark with Ag NPs or Fe_3_O_4_ NPs exhibited the presence of yellow and red cells with higher percentages than the green ones. Interestingly, the cells treated with photothermally active Ag NPs or Fe_3_O_4_ NPs displayed more red cells, up to 68% and 98%, respectively, in comparison to the dark-treated cells group. The appearance of yellow or red colors is related to early and late apoptosis after staining with the PI dye. This reveals the presence of many dead cells with a damaged membrane that led to the entry of the PI stain into cells [43]. It is depicted that the results reveal the robustness of the proposed method. This robustness can be noticed in the detection of the cells in the very low-quality CLSM image captured from “Fe_3_O_4_ NPs.”

## Conclusion

*B. hominis* pathogenicity has been questioned for years owing to its presence in symptomatic and asymptomatic patients. Lately, the increase in multidrug resistance has highlighted the urgent need for new substitute alternative treatments for parasitic infections. PTT is nominated as a new technique that provides an innovative antipathogenic remedy. Herein, we assessed the effect of Ag NPs and Fe_3_O_4_ NPs as sole agents and the combined action of LED on *B. hominis* in vitro. Our work is considered the first report demonstrating that photothermally active nanoparticles can elucidate a significant reduction in *Blastocystis* cyst count. Accordingly, we suggest that the photothermally active NPs can be considered a promising anti-blastocystis agent. Furthermore, we used a process depending on the color features of the live and dead cells, employing superpixels and *k*-means clustering to segment and differentiate the live and dead cells. Nonetheless, to better understand the mechanism of cyst count reduction and whether these photothermally active NPs induce ultrastructural changes to the cysts, further in vivo studies are required. We also endorse the potential role of various photothermally active nanoparticles and their corresponding nanocomposites for better application in clinical antiparasitic photothermal therapy.

## Data Availability

Data will be made available on request.

## Abbreviations

*B. hominis*: *Blastocystis hominis*
NPs: Nanoparticles
Ag NPs: Silver nanoparticles
Cs: Chitosan
PTT: Photothermal therapy
NIR: Near infrared
PEG: Polyethylene glycol
AO: Acridine orange
PI: Propidium iodide
ASLIC: Adaptive simple linear iterative clustering
